# A Conserved Mitochondrial Chaperone-Protease Complex Involved in Protein Homeostasis

**DOI:** 10.3389/fmolb.2021.767088

**Published:** 2021-11-09

**Authors:** Mauro Serricchio, Peter Bütikofer

**Affiliations:** Institute of Biochemistry and Molecular Medicine, University of Bern, Bern, Switzerland

**Keywords:** cardiolipin, stomatin-like protein 2, Yme1, prohibitin, mitochondria, mitochondrial stress response, membrane proteins, trypanosoma

## Abstract

Mitochondria are essential organelles involved in cellular energy production. The inner mitochondrial membrane protein stomatin-like protein 2 (SLP-2) is a member of the SPFH (stomatin, prohibitin, flotilin, and HflK/C) superfamily and binds to the mitochondrial glycerophospholipid cardiolipin, forming cardiolipin-enriched membrane domains to promote the assembly and/or stabilization of protein complexes involved in oxidative phosphorylation. In addition, human SLP-2 anchors a mitochondrial processing complex required for proteolytic regulation of proteins involved in mitochondrial dynamics and quality control. We now show that deletion of the gene encoding the *Trypanosoma brucei* homolog TbSlp2 has no effect on respiratory protein complex stability and mitochondrial functions under normal culture conditions and is dispensable for growth of *T. brucei* parasites. In addition, we demonstrate that TbSlp2 binds to the metalloprotease TbYme1 and together they form a large mitochondrial protein complex. The two proteins negatively regulate each other’s expression levels by accelerating protein turnover. Furthermore, we show that TbYme1 plays a role in heat-stress resistance, as TbYme1 knock-out parasites displayed mitochondrial fragmentation and loss of viability when cultured at elevated temperatures. Unbiased interaction studies uncovered putative TbYme1 substrates, some of which were differentially affected by the absence of TbYme1. Our results support emerging evidence for the presence of mitochondrial quality control pathways in this ancient eukaryote.

## Introduction

Stomatin-like protein 2 (SLP-2) belongs to the SPFH (stomatin, prohibitin, flotilin, and HflK/C) superfamily. Members of the SPFH superfamily contain a conserved Band seven domain and have been shown to cluster and form membrane microdomains that stabilize multiprotein complexes (Rivera-Milla et al., 2006). SLP-2 was described to be overexpressed in numerous cancer types and involved in cancer development and progression (Zhang et al., 2006; Cao et al., 2007; Deng et al., 2017; Liu et al., 2020). SLP-2 localizes to the plasma membrane (Wang and Morrow, 2000; Kirchhof et al., 2008) and mitochondria (Christie et al., 2011), where it binds to the mitochondrial inner membrane through interaction with the glycerophospholipid cardiolipin (CL). In mitochondria, SLP-2 helps to form CL-enriched domains by interacting with prohibitins (PHB1 and PHB2), two additional members of the SPFH superfamily (Christie et al., 2011). In T cells, SLP-2 is important for respiratory supercomplex formation (Mitsopoulos et al., 2015), mitochondrial translation (Mitsopoulos et al., 2017) and the formation of functional plasma membrane microdomains that help assemble T cell receptor components (Christie et al., 2012). SLP-2 also interacts with T cell receptor signalosome components and contributes to sustain T cell activation (Kirchhof et al., 2008). In addition, human SLP-2 anchors a mitochondrial processing complex (“SPY” complex), consisting of SLP-2, PARL and YME1L (Wai et al., 2016), which is required for proteolytic regulation of proteins involved in mitochondrial dynamics and quality control. Within the SPY complex, SLP-2 regulates the activity of the intermembrane space AAA-ATPase YME1L towards proteolytic degradation of specific substrates (Wai et al., 2016). YME1L is a membrane-bound metalloprotease that forms homo-hexamers and is involved in the degradation of unfolded or excess proteins (Shi et al., 2016). Point mutations of human YME1L can cause mitochondriopathy, optic atrophy and mitochondrial fragmentation (Hartmann et al., 2016).

CL is a dimeric mitochondrial glycerophospholipid instrumental for proper functioning of mitochondria. It is tightly associated with respiratory complexes and required for respiratory supercomplex assembly and F_o_F_1_-ATPase dimerization (Acehan et al., 2011). The length and degree of saturation of the four acyl chains of CL are tightly controlled during CL metabolism, and defects in CL fatty acyl chain remodeling result in the human disease Barth syndrome (Schlame and Ren, 2006; Raja and Greenberg, 2014; Zegallai and Hatch, 2021). Barth syndrome patients often suffer from cardiomyopathy, skeletal myopathy, neutropenia and growth retardation. Interestingly, the human enzymes involved in CL biosynthesis, phosphatidylglycerophosphate synthase (PGS1) and cardiolipin synthase (CRLS1), bind to both SLP-2 and PHB1 (Serricchio et al., 2018). A possible functional connection between CL biosynthesis and the formation of CL-enriched microdomains has not been reported.

*Trypanosoma brucei* is a unicellular protozoan parasite causing human African Trypanosomiasis, also known as sleeping sickness, and nagana in domestic animals in Sub-Saharan Africa. *T. brucei* is an established model organism to study eukaryotic cell biology (Bütikofer et al., 2001; Fairlamb et al., 2016; Schneider and Ochsenreiter, 2018) and lipid metabolism (Serricchio and Bütikofer, 2010; Ramakrishnan et al., 2013). This highly diverged eukaryote is unrelated to Opisthokonts (Walker et al., 2011) and thus provides a unique opportunity to study ancestral functions of organelles and proteins (Bütikofer et al., 2001; Schneider, 2018; Schneider and Ochsenreiter, 2018) that are conserved in higher eukaryotes. Two enzymes of the CL biosynthetic pathway have been identified and studied in *T. brucei*, revealing that *T. brucei* phosphatidylglycerophosphate synthase (TbPgs) and *T. brucei* cardiolipin synthase (TbCls) are essential for CL biosynthesis, mitochondrial function and parasite survival (Serricchio and Bütikofer, 2012; 2013).

Here, we identify and characterize the *T. brucei* SLP-2 homolog (TbSlp2) and show that, in contrast to human, it is dispensable for mitochondrial health. TbSlp2 localizes to mitochondria where it binds to membranes via phosphatidic acid (PA) and interacts with prohibitin 1 (TbPhb1) and TbPgs, possibly linking CL biosynthesis to CL microdomain formation. Moreover, we demonstrate that TbSlp2 forms a protozoan “SPY”-like complex with a newly identified *T. brucei* YME1L homolog (TbYme1). Interestingly, TbSlp2 and TbYme1 negatively regulate each other and are involved in mitochondrial stress response by acting as pro-survival proteins.

## Results

### Stomatin-like Protein 2 is Conserved in *Trypanosoma brucei*

**FIGURE 1:**
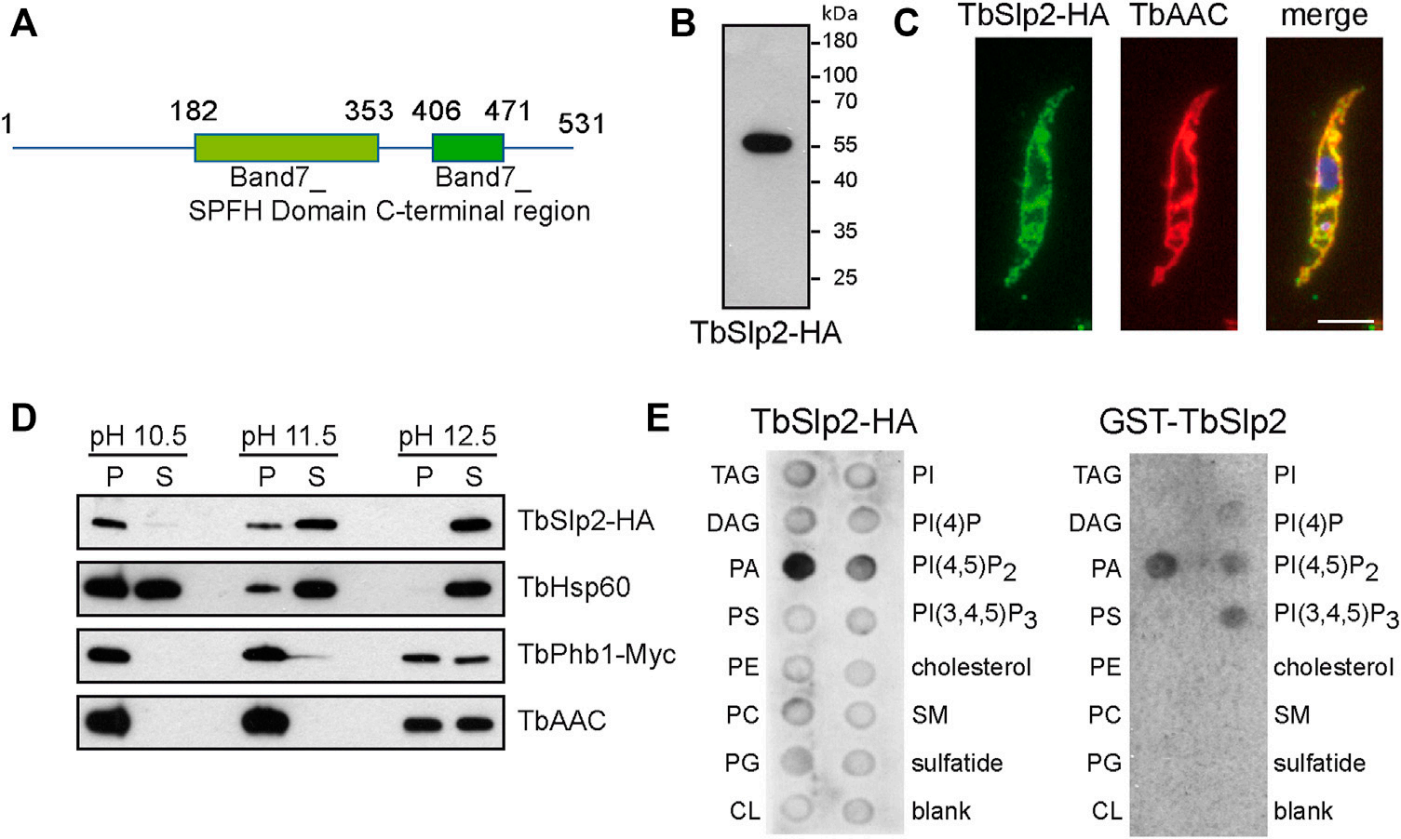
Characterization of TbSlp2 in *T. brucei*. **(A)** Schematic of TbSlp2 showing the location of the conserved Band7_SPFH domain and the C-terminal region with the amino acid numbers indicated. **(B)** SDS-PAGE and immunoblot analysis of TbSlp2-HA expressed in *T. brucei* procyclic forms. **(C)** *T. brucei* procyclic forms expressing TbSlp2-HA were incubated with antibodies against HA and the mitochondrial ADP/ATP carrier (TbAAC) and analyzed by fluorescence microscopy. Scale bar: 5 µm **(D)** Carbonate extraction of TbSlp2-HA. Mitochondrial membranes were treated with 0.1 M Na_2_CO_3_ at pH 10.5, 11.5 and 12.5, separated by ultracentrifugation into soluble (S) and membrane (P) fractions, and analyzed by immunoblotting using primary antibodies against HA, TbHsp60 (soluble matrix protein), or TbPhb1 and TbAAC (integral membrane proteins). **(E)** TbSlp2-HA isolated from *T. brucei* **(left panel)** and recombinant GST-TbSlp2 purified from *E. coli* **(right panel)** was added to membranes containing pre-spotted lipids and protein binding was visualized using antibodies against HA and GST, respectively. TAG: triacylglycerol; DAG: diacylglycerol; PA: phosphatidic acid; PS: phosphatidylserine; PE: phosphatidylethanolamine; PC: phosphatidylcholine; PG: phosphatidylglycerol; CL: cardiolipin; PI: phosphatidylinositol; PI(4)P: phosphatidylinositol 4-phosphate; PI(4,5)P2: phosphatidylinositol 4,5-biphosphate; PI(3,4,5)P3: phosphatidylinositol 3,4,5-triphosphate; SM: sphingomyelin.

The *T. brucei* genome encodes three proteins containing SPFH domains, TbPhb1, TbPhb2 and a putative stomatin-like protein (Tb927.5.520). Blast searches with the putative *T. brucei* stomatin-like protein against the human proteome revealed the most significant alignment with SLP-2/STOML2. Pairwise sequence alignment of the deduced full-length *T. brucei* stomatin-like protein (TbSlp2) with human SLP-2 revealed an overall sequence identity of 30% (41% sequence similarity). The stomatin domain alone revealed a 57% sequence identity and a 74% sequence similarity with human SLP-2. The deduced TbSlp2 protein has a calculated molecular mass of 56 kDa, contains a conserved SPFH domain and a C-terminal domain, but lacks transmembrane domains or membrane hairpins (Lapatsina et al., 2012) (Figure 1A).

To study TbSlp2, we generated *T. brucei* procyclic forms expressing C-terminally HA-tagged TbSlp2 from their genomic locus (henceforth called *in situ* tagged TbSlp2-HA) and analyzed total parasite protein by SDS-PAGE and immunoblotting. The results show that TbSlp2-HA migrated as a single ∼55 kDa band (Figure 1B). Subsequently, the subcellular localization of TbSlp2-HA was studied using immunofluorescence microscopy. Co-staining of parasites expressing TbSlp2-HA using antibodies against HA and the mitochondrial ADP/ATP carrier (TbAAC) revealed that TbSlp2-HA localizes to the mitochondrion (Figure 1C).

Members of the SPFH family are membrane-associated proteins that form ring-like complexes involved in compartmentalization of the inner mitochondrial membrane (Langhorst et al., 2005; Browman et al., 2007). TbSlp2, however, has no predicted transmembrane domain or predicted lipid modification that would indicate membrane association. To study if TbSlp2-HA is membrane-associated, we used carbonate extraction at different pH to test its affinity to mitochondrial membranes. The results show that TbSlp2-HA is firmly membrane-associated at pH 10.5, but soluble at pH 12.5 (Figure 1D). At pH 11.5, TbSlp2-HA is present in both fractions. A similar behavior at pH 11.5 and pH 12.5 was also observed for the mitochondrial matrix chaperone TbHsp60 (Figure 1D). In contrast, the two membrane-integral proteins TbPhb1 and TbAAC are completely membrane-associated at pH 10.5 and 11.5 and partly membrane-associated even at pH 12.5 (Figure 1D).

We next studied a possible binding affinity of TbSlp2 towards different glycerophospholipids using a lipid overlay experiment. TbSlp2 was either purified by immunoprecipitation from *T. brucei* expressing *in situ* tagged TbSlp2-HA or by affinity chromatography from *E. coli* expressing recombinant glutathione-S-transferase (GST)-conjugated TbSlp2 (GST-TbSlp2). Incubation of glycerophospholipid-coated membranes with purified TbSlp2-HA or GST-TbSlp2 and subsequent detection with anti-HA or anti-GST antibodies revealed that TbSlp2 interacts strongly with PA, and weakly with phosphoinositides, while no interactions with CL or other phospholipids were observed (Figure 1E).

### *T. brucei* Stomatin-like Protein 2Slp2 is Dispensable for Growth of Procyclic Parasites

To study the role of TbSlp2 in *T. brucei* procyclic forms, we used stably Cas9-expressing SmOx P9 parasites (Beneke et al., 2017) to knock-out gene Tb927.5.520 by sgDNA-targeted ssDNA nicking near the 5′- and 3′-ends of the open reading frame (ORF) (Supplementary Figure S1A). Providing a repair template containing a G418 resistance cassette flanked by 30 nucleotide homology regions matching the untranslated regions adjacent to the open reading frame, we were able to generate TbSlp2 knock-out (TbSlp2−/−) parasites using a single resistance cassette. Analyzing individual G418-resistant clones by PCR with different combinations of gene-specific primers, we identified clones that integrated the resistance gene into one allele (TbSlp2^+/−^) or two alleles simultaneously (TbSlp−/−) (Supplementary Figure S1B). When we analyzed cell proliferation, we found a slightly reduced growth rate of TbSlp2−/− parasites (doubling time 12.2 ± 1.2 h) as compared to the wild-type cells (doubling time 11.5 ± 0.8 h). These results show that TbSlp2 is dispensable for survival in culture under ideal growth conditions.

### *T. brucei* Stomatin-like Protein 2 Deficiency Does Not Affect Mitochondrial Function

In SLP2-deficient mouse T cells, the mitochondrial membrane potential was shown to be decreased and formation of respiratory chain supercomplexes was affected (Mitsopoulos et al., 2015). To study possible effects of TbSlp2 depletion on mitochondrial function, we examined the mitochondrial membrane potential ΔΨm in *T. brucei* wild-type and TbSlp2−/− parasites grown in high- or low-glucose medium. In low-glucose medium, *T. brucei* procyclic forms depend primarily on mitochondrial amino acid metabolism for ATP production as compared to glycolysis in high-glucose conditions (Lamour et al., 2005). Our results show that ΔΨm-dependent uptake of tetramethylrhodamine ethylester (TMRE) *in vivo* was not affected by depletion of TbSlp2, irrespective of the culture medium used (Supplementary Figure S1C). In line with these results, we detected no differences between wild-type and TbSlp2−/− parasites in the size and stability of the respiratory complexes IV (detected using anti-Cox4 antibody) and III (detected using anti-Cyc c1 antibody), and the migration of TbAAC after native gel electrophoresis (native PAGE) (Supplementary Figure S1D). As additional readout for mitochondrial health, we measured oxygen consumption rates using Seahorse flux analyser. We found that basal respiration, maximal respiration, spare respiratory capacity and non-mitochondrial respiration were not significantly different between wild-type and TbSlp2−/− parasites (Supplementary Figure S1E). Finally, using immunofluorescence microscopy we observed similar reticulate mitochondrial staining patterns of TbAAC in wild-type and TbSlp2−/− parasites (Supplementary Figure S1F). Together, these results show that under standard culture conditions, TbSlp2 is not required to maintain mitochondrial integrity and function in *T. brucei* procyclic forms.

### *T. brucei* Stomatin-like Protein 2 interacts With TbPhb1 and TbPgs

**FIGURE 2:**
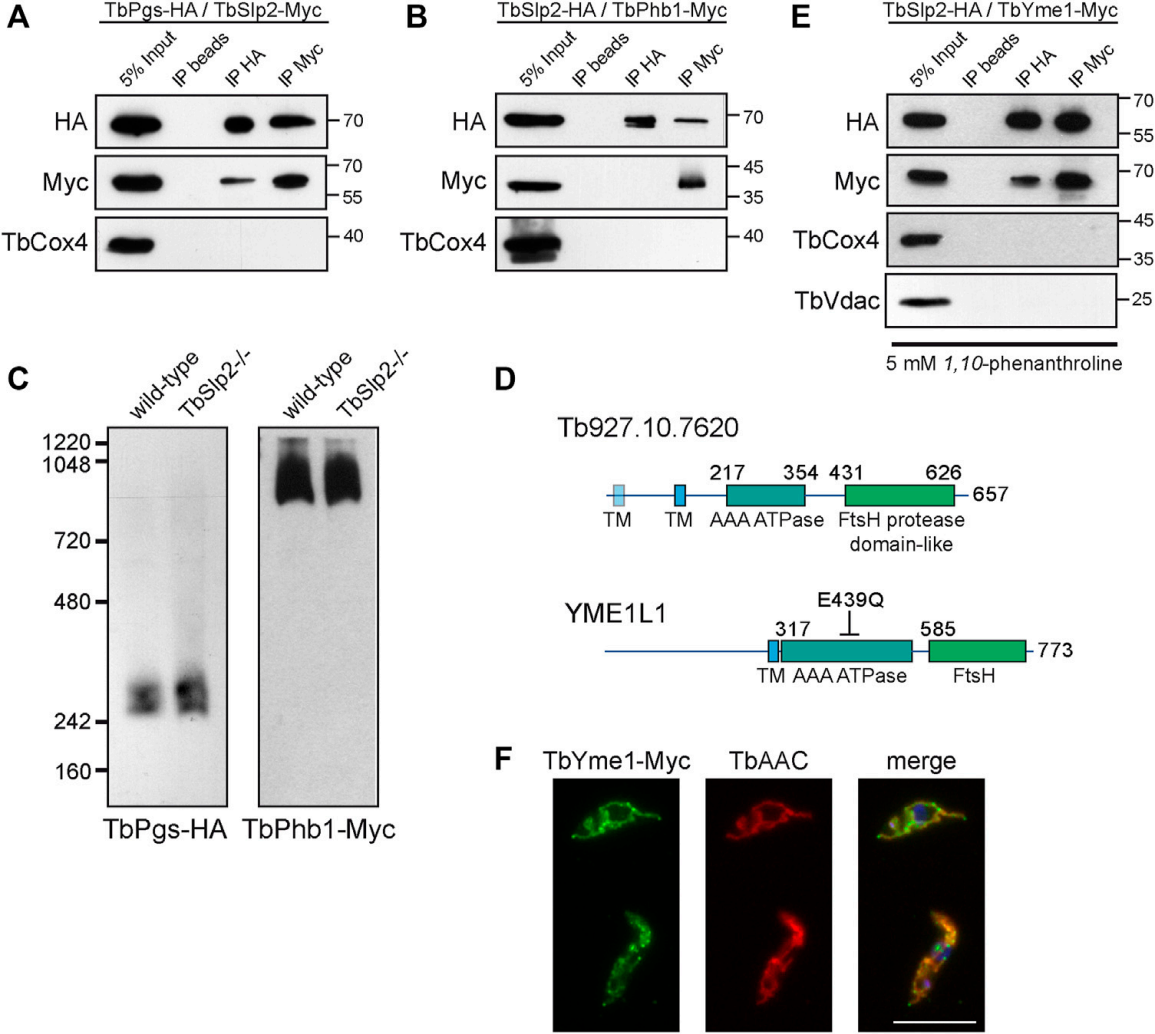
TbSlp2 interacts with the AAA-ATPase TbYme1. **(A)** Co-expressed TbSlp2-Myc and TbPgs-HA were immunoprecipitated with HA or Myc antibodies, or with beads alone, and analyzed by SDS-PAGE and immunoblotting. **(B)** Co-expressed TbSlp2-Myc and TbPhb1-HA were immunoprecipitated with HA or Myc antibodies, or beads alone, and analyzed by immunoblotting. TbCox4 served as a control protein that should not co-purify. **(C)** TbPgs-HA **(left panel)** and TbPhb1-Myc **(right panel)** expressed in wild-type or TbSlp2−/− parasites were analyzed by native PAGE and immunoblotting using HA or Myc antibodies. **(D)** Schematic representation of the domain structure of uncharacterized protein Tb927.10.7620 in comparison to human YME1L1. The mutation E439Q in the ATPase domain of human YME1L1 leads to loss of ATPase activity. The first transmembrane domain (TM) in Tb927.10.7620 was identified experimentally by (Kovalinka et al., 2020). **(E)** TbSlp2-HA co-expressed with TbYme1-Myc were immunoprecipitated using beads alone or HA and Myc antibodies in the presence of 5 mM 1, 10-phenanthroline. Proteins were separated by SDS-PAGE and analyzed by immunoblotting. TbCox4 and TbVdac were used as to control for unspecific binding. **(F)** The sub-cellular localization of *in situ*-tagged TbYme1-Myc was analyzed by immunofluorescence microscopy using the TbAAC antibody as a marker for the mitochondrial inner membrane. Scale bar: 10 µm.

To study possible interactions between TbSlp2 and proteins involved in CL biosynthesis and binding, we co-expressed *in situ* tagged TbSlp2 with *in situ* tagged TbPgs or TbPhb1 in *T. brucei* procyclic forms and performed reciprocal immunoprecipitation experiments. The results show that TbSlp2 co-precipitated with both TbPgs-HA (Figure 2A) and TbPhb1-Myc (Figure 2B). The reciprocal experiments show that TbSlp2 was able to co-precipitate TbPgs-HA (Figure 2A), while it failed to co-precipitate TbPhb1-Myc (Figure 2B). Since TbPgs (Serricchio and Bütikofer, 2013) and TbPhb1 (Tyc et al., 2010) have been shown to form mitochondrial high molecular mass complexes, we wondered if TbSlp2 plays a role in the formation and/or stabilization of these complexes. We therefore *in situ* tagged TbPgs and TbPhb1 in wild-type and TbSlp2−/− parasites and analyzed mitochondrial protein complexes by native PAGE and immunoblotting. Our results show that in wild type cells TbPgs-HA migrated as a doublet at ∼300 kDa, whereas TbPhb1-Myc was detected as a broad band migrating at ∼1 MDa (Figure 2C). The sizes and intensities of these complexes were unchanged in TbSlp2−/− parasites (Figure 2C), indicating that TbSlp2 is not involved in their assembly or stability. In addition, using metabolic labeling experiments with [^3^H]-glycerol and analysis of [^3^H]-labeled lipid extracts by thin-layer chromatography and radioisotope scanning, we found no differences in *de novo* CL or phosphatidylglycerol formation between wild-type and TbSlp2−/− parasites (Supplementary Figure S2), indicating that TbSlp2 is not involved in CL biosynthesis.

### *T. brucei* Stomatin-like Protein 2 interacts With the Conserved Protease TbYme1

To identify additional interaction partners of TbSlp2, we used an unbiased proteomic approach by immunoprecipitating *in situ* tagged TbSlp2-HA and analyzing co-precipitated proteins by mass spectrometry. Two different extracts were prepared for analysis: 1) total protein from parasites lysed with Triton X-100 and NP-40, and 2) crude mitochondrial membranes solubilized with n-dodecyl-maltoside (DDM). Our analyses revealed three proteins that were precipitated in TbSlp2-HA expressing parasites but absent in control untagged cells, independent of sample preparation: 1) the bait TbSlp2, 2) a protein annotated as putative mitochondrial ATP-dependent zinc metallopeptidase (Tb927.10.7620) and 3) mitochondria-localized TbHsp60 (Tb927.10.6510). Sequence comparisons indicated that Tb927.10.7620 is a member of the FtsH protease family with a high degree of homology to human YME1L (30% sequence identity, 42% similarity). The deduced protein contains one, or possibly two, transmembrane domains (Figure 2D) that anchor it to the mitochondrial inner membrane, facing the mitochondrial matrix (Kovalinka et al., 2020) (see below); we have re-named this protein TbYme1. To confirm the interaction between TbSlp2 and TbYme1, we generated double *in situ* tagged *T. brucei* procyclic forms and performed reciprocal co-immunoprecipitation experiments. The results show that TbSlp2-HA co-precipitated TbYme1-Myc, and vice versa (Figure 2E), indicating their tight interaction. To prevent rapid degradation of TbYme1-Myc during the immunoprecipitation experiments, the metalloprotease inhibitor *1,10*-phenanthroline was added to the lysis buffer. Immunofluorescence microscopy of *in situ* tagged TbYme1-Myc and co-staining with TbAAC confirmed that TbYme1 is a mitochondrial protein (Figure 2F).

### TbYme1 and *T. brucei* Stomatin-like Protein 2 Form a Large Mitochondrial Complex

**FIGURE 3:**
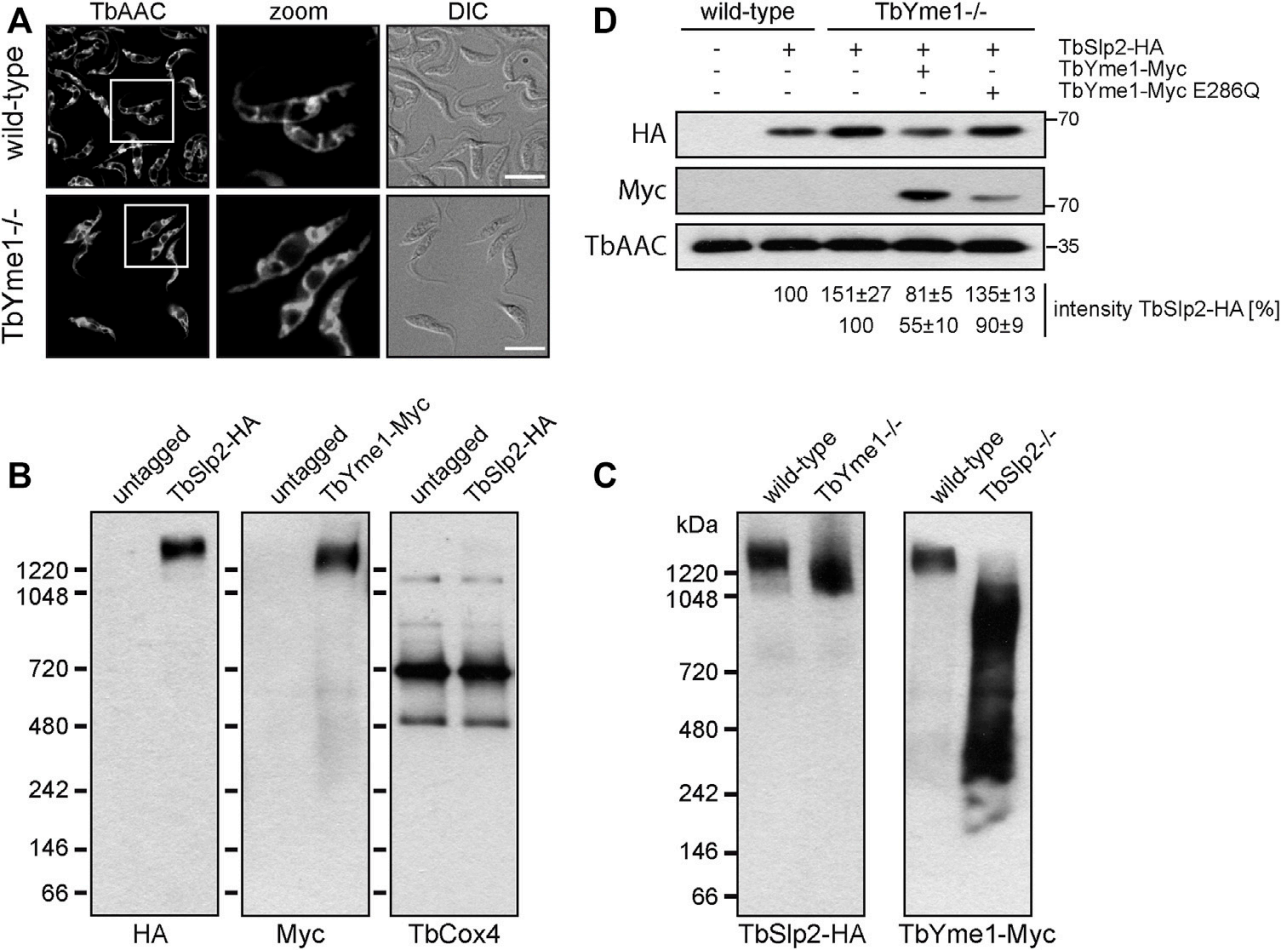
TbSlp2 and TbYme1 form a large complex. **(A)** Mitochondrial integrity of wild-type and TbYme1−/− parasites was assessed by immunofluorescence microscopy using an antibody against TbAAC. The white box marks the area that is enlarged in the second panel. Scale bar: 10 µm. **(B)** Native-PAGE and immunoblot analysis of *in situ*-tagged TbSlp2-HA or TbYme1-Myc expressed in wild-type *T. brucei.* Untagged parasites were used to control for antibody specificity, and TbCox4 was used as a loading control. **(C)** TbSlp2-HA expressed in wild-type and TbYme1−/− parasites **(left panel)** or TbYme1-Myc expressed in wild-type and TbSlp2−/− parasites **(right panel)** were analysed by native-PAGE and immunoblotting using HA or Myc antibodies, respectively. **(D)** Quantification by immunoblotting of TbSlp2-HA expressed in wild-type, TbYme1−/− or TbYme1−/− parasites re-expressing TbYme1-Myc or TbYme1-Myc E286Q. Proteins were quantified relative to TbAAC and intensity values are given below the blot (n ≥ 3).

To study the role of TbYme1 in *T. brucei*, we used CRISPR/Cas9 to replace the TbYme1 ORF sequentially with two resistance cassettes (Supplementary Figure S3A). PCR was performed to confirm the absence of the TbYme1 ORF from clones obtained after first-allele and second-allele replacement (Supplementary Figure S3B). TbYme1−/− parasites were viable in culture but grew slower than parental parasites, with cell doubling times of 14.3 ± 0.6 h as compared to 9.8 ± 0.2 h. Analysis of respiratory chain complexes by native PAGE and oxygen consumption by Seahorse flux analysis revealed no significant differences between TbYme1−/− and parental parasites (Supplementary Figure S3C–E). Loss of TbYme1 had no effect on mitochondrial morphology, as immunofluorescence staining of TbAAC in wild-type and TbYme1−/− parasites appeared similar (Figure 3A).

It has been shown that human SLP-2 is part of a mitochondrial complex migrating at ∼2 MDa (Wai et al., 2016). To test if TbSlp2 migrates as a complex in *T. brucei*, we separated DDM-solubilized mitochondrial membranes by native PAGE. Detection of *in situ* tagged TbSlp2-HA by immunoblotting revealed a large complex of ∼1.5 MDa (Figure 3B). A complex of similar size was detected when analyzing the migration of TbYme1-Myc (Figure 3B), providing additional evidence that the two proteins interact with each other (see also Figure 2E). We then studied the formation and/or stability of the TbSlp2-TbYme1 complex in parasites lacking either protein. For this we expressed TbSlp2-HA in TbYme1−/− parasites and, conversely, TbYme1-Myc in TbSlp2−/− parasites. Analysis by native PAGE and immunoblotting revealed that the ∼1.5 MDa complex containing TbSlp2-HA in parental cells was shifted downwards to ∼1.1 MDa in parasites lacking TbYme1 (Figure 3C). Unexpectedly, analysis of TbYme1-Myc revealed that the ∼1.5 MDa complex containing TbYme1-Myc in parental cells was absent in TbSlp2−/− parasites (Figure 3C). Instead TbYme1-Myc migrated as a broad band in the molecular mass range 250 kDa - 1 MDa (Figure 3C).

To examine a possible role of TbYme1 on TbSlp2 expression, we then compared TbSlp2 protein levels between wild-type and TbYme1−/− parasites. Analysis by SDS-PAGE followed by protein quantification showed an increase in TbSlp2-HA levels by ∼50% in TbYme1−/− parasites as compared to wild-type cells (Figure 3D). In control cells expressing TbYme1-Myc in the TbYme1−/− background, TbSlp2-HA levels were reduced to ∼80% of wild-type levels, or to 55% of the levels in parental TbYme1−/− parasites (Figure 3D). We next expressed a mutant form of TbYme1, TbYme1-Myc E286Q, containing a point mutation in the predicted catalytic site rendering it catalytically inactive (Hartmann et al., 2016; Shi et al., 2016). TbSlp2-HA levels again increased to 135% compared to wild-type cells, or to 90% compared to parental TbYme1−/− parasites (Figure 3D), however, it should be noted that expression levels of TbYme1-E286Q were consistently lower when compared to wild-type TbYme1-Myc. Together, these results indicate that TbSlp2 expression is regulated by the presence of TbYme1.

### TbYme1 and *T. brucei* Stomatin-like Protein 2 Negatively Regulate Each Other

**FIGURE 4:**
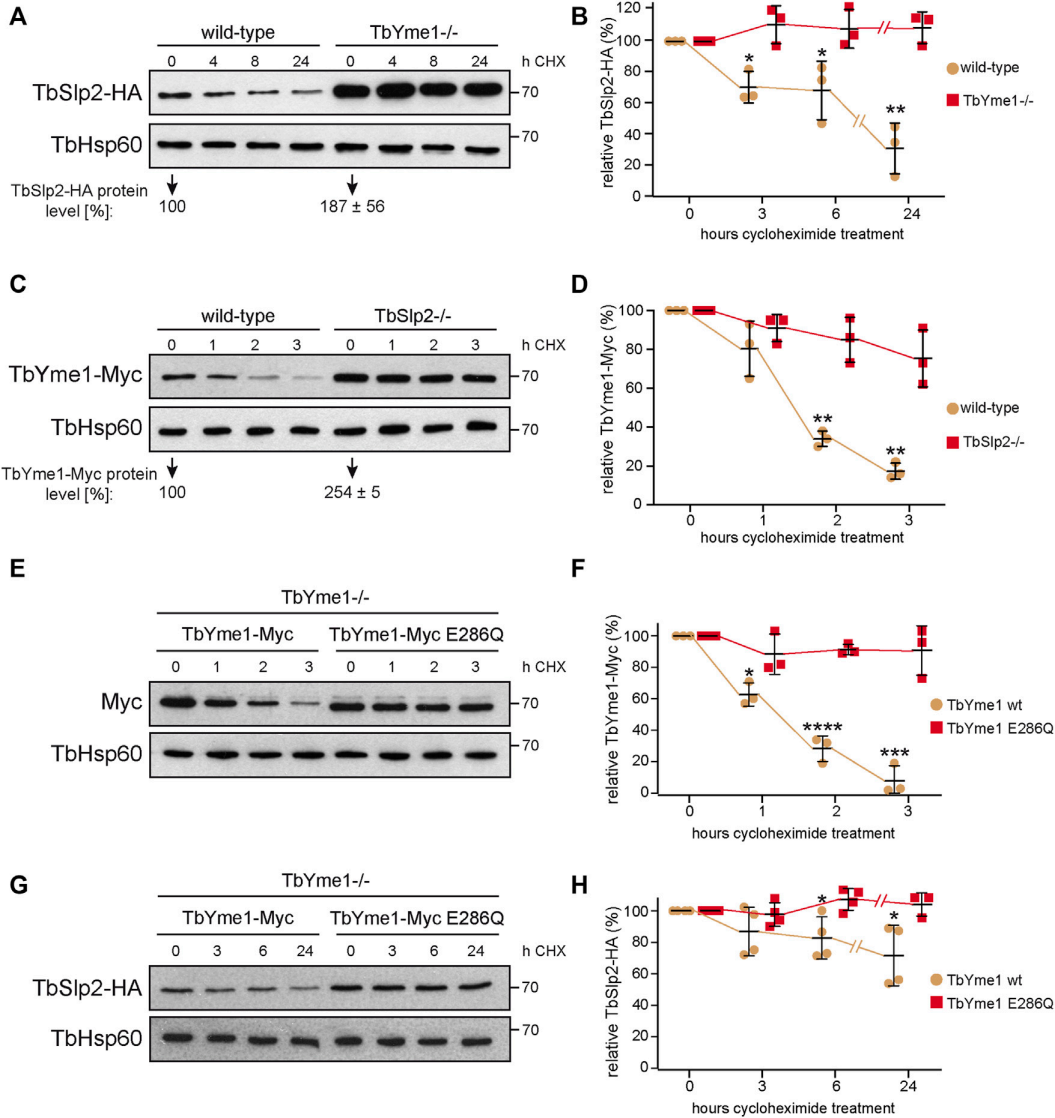
TbSlp2 and TbYme1 are interdependent. **(A)** Wild-type and TbYme1−/− *T. brucei* expressing TbSlp2-HA *in situ* were treated with cycloheximide (CHX) for the times indicated. Proteins were separated by SDS-PAGE and analysed by immunoblotting. TbHsp60 was used as a loading control. Normalized TbSlp2-HA protein levels in untreated cells are given below the immunoblots (n ≥ 3). **(B)** Quantification of TbSlp2-HA signal intensity normalized to TbHsp60 from data shown in A) (n ≥ 3). **(C)** Wild-type and TbSlp2−/− parasites expressing TbYme1-Myc were treated with CHX for the times indicated. Proteins were separated by SDS-PAGE and analysed by immunoblotting. Normalized TbYme1-Myc protein levels in untreated cells are given below the immunoblots (n ≥ 3). **(D)** Quantification of TbYme1-Myc signal intensities normalized to TbHsp60 from images depicted in C). **(E)** Turnover of TbYme1-Myc or TbYme1-Myc E286Q stably expressed in TbYme1−/− parasites was analysed after CHX treatment for times indicated. **(F)** Quantification of TbYme1-Myc or TbYme1-Myc E286Q signal intensities normalized to TbHsp60 as shown in E). **(G)** Turnover of TbSlp2-HA after CHX treatment of TbYme1−/− parasites co-expressing TbYme1-Myc or TbYme1-Myc E286Q. **(H)** Quantification of TbSlp2-HA protein levels normalized to TbHsp60 from images shown in G). Students t-test; *: *p* < 0.05; **: *p* < 0.01; ***: *p* < 0.005; ****: *p* < 0.0005.

To study if TbYme1 and TbSlp2 affect each other`s stability, we followed the turnover of *in situ* tagged TbSlp2-HA expressed in parental or TbYme1−/− parasites after cycloheximide treatment. In parental cells, TbSlp2-HA had a turnover rate t_1/2_ of ∼15 h, while in TbYme1−/− cells, TbSlp2-HA was stable for at least 24 h (Figures 4A,B). These results are in line with our findings (see Figure 3D) that TbSlp2-HA protein levels are approximately two-fold up-regulated in TbYme1−/− parasites and the turnover of TbSlp2-HA is attenuated in TbYme1−/− cells. Next, we analyzed the stability of *in situ* tagged TbYme1-Myc in wild-type and TbSlp2−/− parasites and found that the level of TbYme1-Myc in TbSlp2−/− parasites was 2.5-fold higher than in wild-type cells (Figure 4C). In addition, our results show that the turnover of TbYme1-Myc in TbSlp2−/− parasites (t_1/2_ ≈ 6.3 h) was significantly decreased compared to wild-type cells (t_1/2_ ≈ 1.8 h) (Figure 4D).

Since TbYme1 is a putative metalloprotease and is unstable during parasite lysis (as mentioned above), we studied if it may undergo autocatalytic degradation. We expressed wild-type and the catalytically inactive TbYme1-E286Q variant in TbYme1−/− parasites and quantified protein levels after cycloheximide treatment. We noted a small downward size-shift in TbYme1-E286Q compared to the wild-type protein, possibly due to the change in acidity (Figure 4E). The results show that wild-type TbYme1-Myc was degraded quickly, while the mutant TbYme1-E286Q remained stable (Figures 4E,F), indicating that TbYme1 undergoes autocatalytic processing. In addition, we found that the turnover of TbSlp2-HA was completely blocked in TbYme1−/− parasites expressing the catalytically inactive TbYme1-E286Q variant (Figures 4G,H), indicating that the turnover/degradation of TbSlp2-HA is regulated by TbYme1-Myc.

### *T. brucei* Stomatin-like Protein 2 and TbYme1 Are important for Mitochondrial Quality Control

**FIGURE 5:**
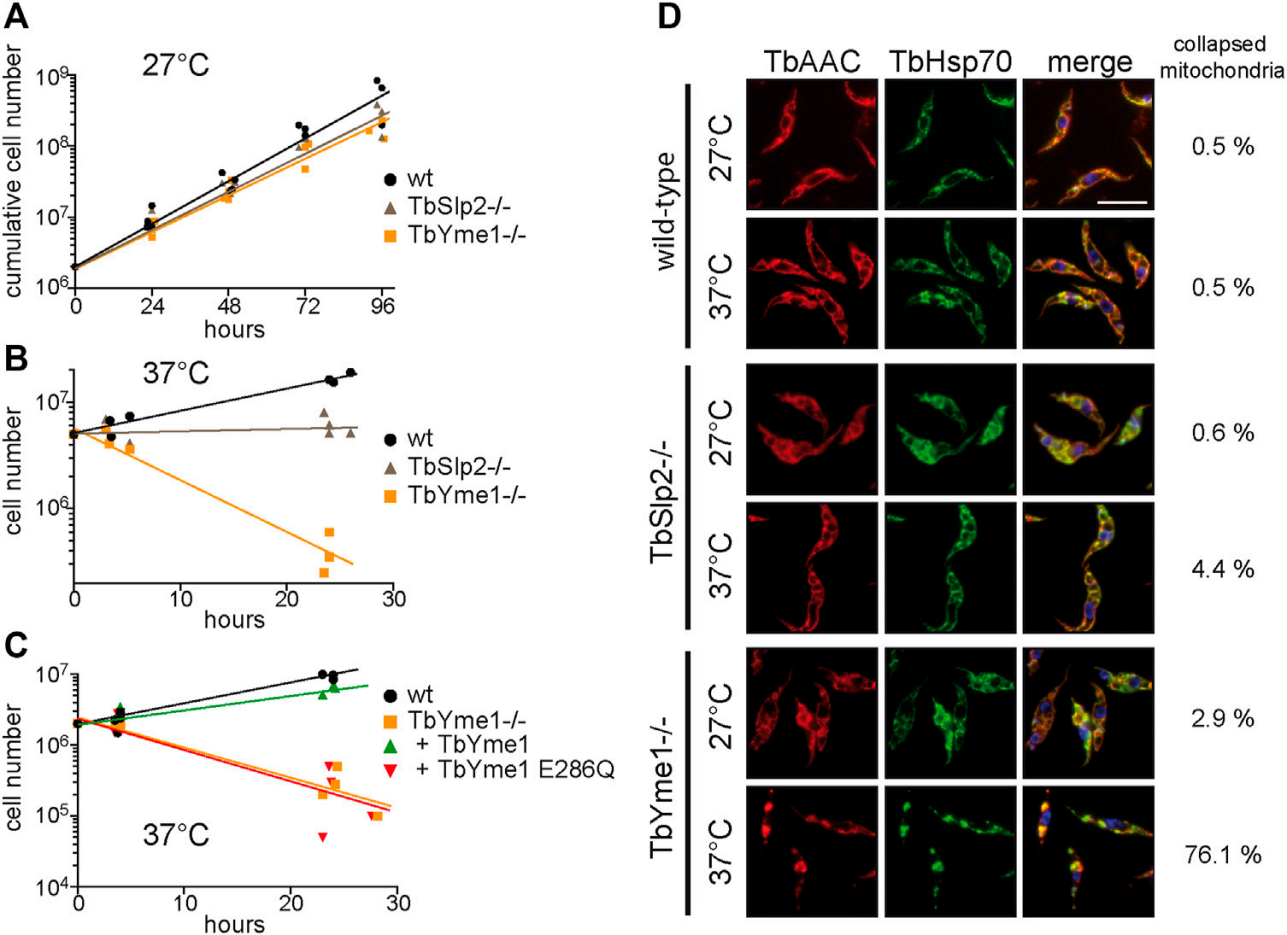
TbYme1 is required for heat stress tolerance. **(A)** Growth curves of wild-type, TbSlp2−/− and TbYme1−/− parasites cultured at 27°C. **(B)** Cell numbers of wild-type, TbSlp2−/− and TbYme1−/− parasites cultured at 37°C for a total period of 24 h. **(C)** Wild-type, TbYme1−/− and TbYme1−/− parasites re-expressing TbYme1 or TbYme1 E286Q were diluted to the same density and cultured at 37°C. Cell numbers were determined at different time points. **(D)** Mitochondrial morphology of wild-type, TbSlp2−/− and TbYme1−/− parasites was assessed by immunofluorescence microscopy using TbAAC and TbHsp70 antibodies. Parasites were cultured at 27°C or at 37°C for 4 h, fixed and processed for microscopy. Mitochondria were visually categorized as reticulate or collapsed based on the appearance of large mitochondrial aggregates. Numbers of parasites with collapsed mitochondria are given next to the images (n > 100). Scale bar: 10 µm.

Mitochondrial proteases are involved in many processes including the unfolded protein response (Pickles et al., 2018; Vogtle, 2021). To investigate a possible role for TbSlp2 and TbYme1 during stress, we determined parasite proliferation at elevated culture temperature. At the standard culture temperature of 27°C, growth of TbSlp2−/− and TbYme1−/− was only slightly reduced (Figure 5A; see above). In contrast, at 37°C only wild-type parasites were able to proliferate, while the density of TbSlp2−/− parasites remained constant and that of TbYme1−/− parasites dropped substantially (Figure 5B). Examination by light microscopy revealed that TbSlp2−/− parasites were mobile and morphologically unaffected, while TbYme1−/− parasites were immobile and appeared dead after 24 h of culture at 37°C. To further substantiate the role of TbYme1 in heat tolerance, we compared growth at 37°C of TbYme1−/− parasites complemented with wild-type or the catalytically inactive TbYme1-Myc variant. The results show that expression of wild-type TbYme1 in TbYme1−/− parasites restored heat tolerance, while the inactive TbYme1-E286Q variant was unable to support growth at 37°C (Figure 5C).

To assess mitochondrial integrity at elevated temperature, parasites were cultured at 27°C and after a heat-pulse (4 h at 37°C) stained with antibodies against the mitochondrial marker proteins TbAAC and TbHsp70. Examination by fluorescence microscopy revealed that the mitochondria of wild-type parasites were not visibly affected by heat stress (<0.5% of mitochondria fragmented at 37°C) (Figure 5D). In contrast, the majority of the mitochondria of TbYme1−/− parasites appeared fragmented (>76%), while the mitochondria of TbSlp2−/− parasites were still reticulated (<4% of mitochondria fragmented) (Figure 5D).

Finally, since heat stress is known to induce oxidative damage (Slimen et al., 2014), we studied if the absence TbYme1 may increase oxidative stress. Quantification of the amount of reactive oxygen species (ROS; hydroxyl, peroxyl and other reactive oxygen species) using dichlorodihydrofluorescein diacetate (Kalyanaraman et al., 2012) revealed that although ROS levels were increased after a heat-shock for 2 h at 37°C (Supplementary Figure S4), as expected, no differences between wild-type and TbYme1−/− parasites were observed at normal or elevated temperatures (Supplementary Figure S4).

### TbYme1 has Differential Effects on Proteins

**FIGURE 6:**
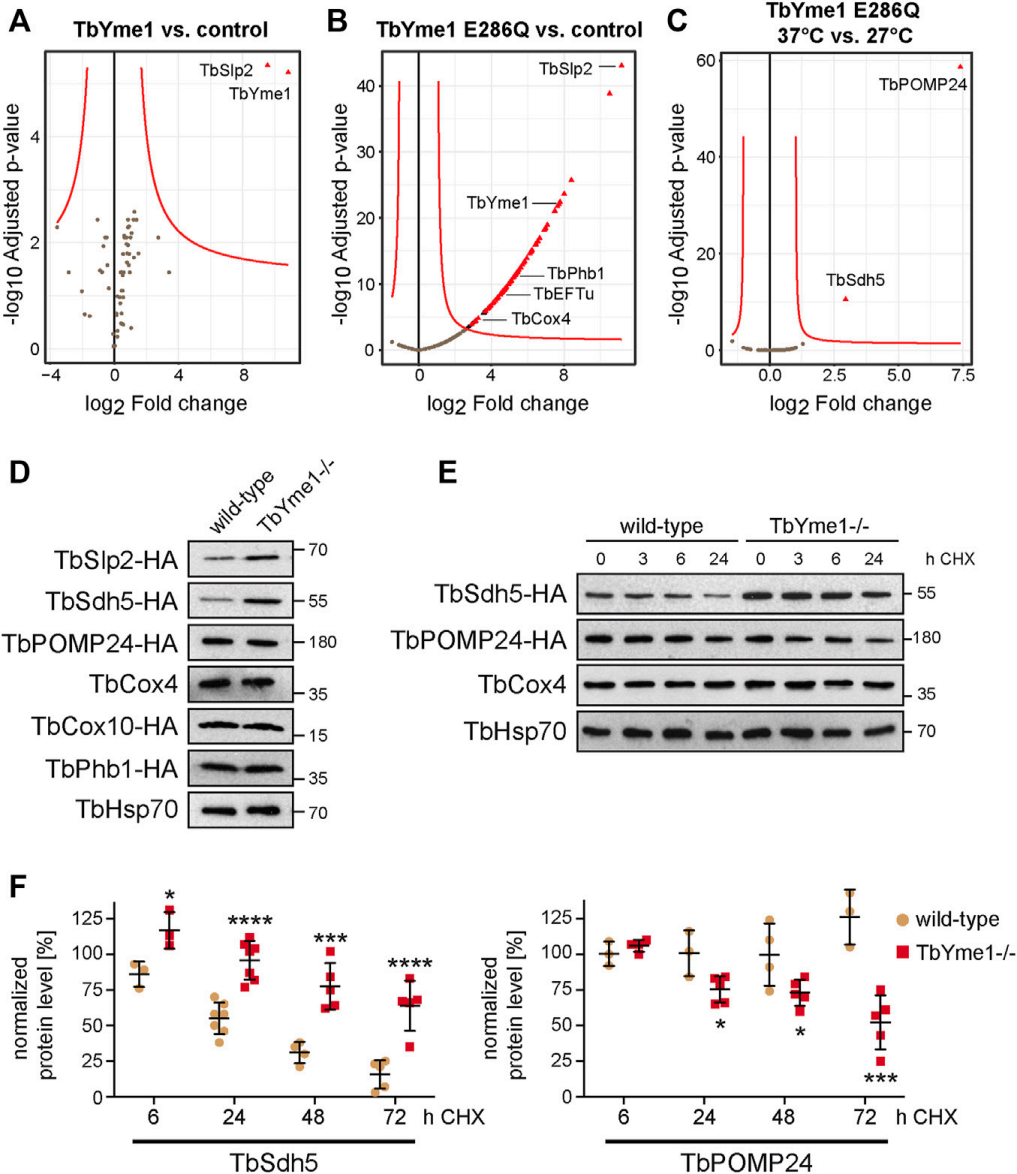
TbYme1 differentially affects its binding partners. Volcano plot of proteins that were enriched after immunoprecipitation of TbYme1-Myc **(A)**, TbYme1-Myc E286Q at 27°C **(B)** and after a heat-pulse of 37°C for 2 h **(C)** compared to parental untagged cells. Cut-off was set to 0.05 < p with a fold-change > 2.5 (red line). Results from A) and C) depict results of duplicate biological replicates, while B) was done in triplicate. **(D)** Putative TbYme1-interacting proteins were *in situ* HA-tagged in wild-type and TbYme1−/− parasites and the protein levels were analysed by SDS-PAGE and immunoblotting. **(E)** Protein analysis by SDS-PAGE and immunoblotting after cycloheximide treatment of wild-type and TbYme1−/− parasites expressing TbSdh5-HA or TbPOMP24-HA. **(F)** Quantification of the protein levels of TbSdh5-HA (left panel) and TbPOMP24-HA (right panel) after 6, 24, 48 and 72 h of cycloheximide treatment from the experiment shown in E). Values represent signal intensities normalized to TbHsp70. Students t-test; *: *p* < 0.05; ***: *p* < 0.005; ****: *p* < 0.0005.

To identify additional potential substrates of TbYme1 we immunoprecipitated TbYme1-Myc expressed in TbYme1−/− parasites and identified co-purified proteins by mass spectrometry. In a first attempt, using wild-type TbYme1-Myc as bait, TbSlp2 was the only protein that was specifically precipitated (Figure 6A), confirming the previous immunoprecipitation using TbYme1-Myc as bait and the results showing that TbSlp2 and TbYme1 interact with each other (see Figure 2E). In a second attempt, we took advantage of a previous observation showing that proteolytically inactive human Yme1-E286Q was able to interact with its substrates (Stiburek et al., 2012). We therefore used TbYme1-E286Q expressed in TbYme1−/− parasites as bait to co-precipitate and identify potential substrates (Figure 6B). The results show that the catalytically inactive TbYme1-E286Q variant pulled down a large number of proteins, including TbSlp2, TbPhb1 and TbPhb2, TbMSP-B, multiple known mitochondrial proteins (elongation factor Tu; TbCOX4, TbCOX10, TbMCP13, among others) and several hypothetical, i.e. uncharacterized, proteins (Supplementary Table S1). Interestingly, when we repeated the experiment after a 2 h heat pulse at 37°C, two proteins, TbPOMP24 (present in the outer mitochondrial membrane proteome 24) and succinate dehydrogenase flavoprotein subunit 5 (TbShd5), were specifically precipitated compared to the pull-down done at 27°C (Figure 6C). We then *in situ* tagged a subset of the proteins pulled down using TbYme1-E286Q to compare their expression levels between wild-type and TbYme1−/− parasites. The results show that, in addition to TbSlp2 (see also Figure 3D), TbSdh5 was clearly more abundant in TbYme1−/− parasites compared to wild-type cells (Figure 6D). Quantification of protein levels revealed that TbSdh5 was increased to 184 ± 16% in TbYme1 −/− parasites compared to wild-type cells. Analysis of protein turnover using cycloheximide treatment demonstrated that TbSdh5-HA, similar to TbSlp2-HA (see Figure 4A), was more stable in TbYme1−/− parasites compared to wild-type cells (Figures 6E,F). In contrast, TbPOMP24-HA showed a faster turnover in the absence of TbYme1 (Figures 6E,F). Together these results indicate that TbYme1, or the TbYme1/TbSlp2 complex, may have different effects on mitochondrial proteins.

## Discussion

In human cells, SLP-2, PARL and YME1L1 form the “SPY complex” that is involved in regulating mitochondrial dynamics and exhibiting anti-apoptotic functions by allowing stress-induced mitochondrial hyperfusion (Tondera et al., 2009; Wai et al., 2016). Human YME1L also controls the accumulation of respiratory chain subunits and is required for apoptotic resistance, cristae morphogenesis, and cell proliferation by degrading its substrates Ndufb6, ND1, and Cox4 (Stiburek et al., 2012). In yeast, a mitochondrial AAA protease complex consisting of Yme1 and the adaptors Mgr1 and Mgr3 is involved in a mitochondrial outer membrane quality control pathway by degrading the outer membrane proteins Tom22 and Om45 (Wu et al., 2018). We have discovered a similar “SPY-like complex” in *T. brucei*, with TbSlp2 forming a complex with TbYme1. Depletion of TbYme1 had no effect on mitochondrial morphology, which contrasts observations in mouse (Ruan et al., 2013) or human (Hartmann et al., 2016) fibroblasts, where YME1L depletion resulted in mitochondrial fragmentation. Interestingly, we found that TbSlp2 and TbYme1 regulate each other’s expression levels. To our knowledge, such an interdependence of components of a mitochondrial proteolytic complex has not been described before. Loss of either TbSlp2 or TbYme1 significantly up-regulated the abundance of the other protein. In time course experiments using cycloheximide, we found a significantly slower turnover of TbSlp2 in the absence of TbYme1, and a slower turnover of TbYme1 in the absence of TbSlp2, indicating that the steady-state levels of TbSlp2 and TbYme1 are regulated at the level of protein stability rather than via protein expression. However, the exact mechanism how the two proteins control each other’s stability, i.e. degradation, remains unclear. Since TbYme1 has been shown to form hexameric structures (Shi et al., 2016), we speculate that TbSlp2 may promote TbYme1 oligomerization within the *T. brucei* complex, causing increased auto-catalytic processing of TbYme1 in *trans*, while in the absence of TbSlp2, TbYme1 may be distributed more diffusely in the membrane, resulting in reduced degradation.

The membrane topology of TbYme1 was characterized previously (Kovalinka et al., 2020)*. In silico* analyses provided conflicting results on the number of transmembrane domains (TMs): nine programs predicted one TM, and six predicted two or more TMs. Using digitonin fractionation and protease digestion experiments, Kovalinska *et al.* experimentally verified that TbYme1 has the same membrane topology as *m*-AAA proteases containing 2TMs and thus face the mitochondrial matrix. Phylogenetic analyses also suggest that TM duplication may have occurred in *T. brucei,* leading to the reverse orientation compared to the orientations of its homologs in yeast (Yme1) (Weber et al., 1996) and plants (FtsH4 and FtsH11) (Urantowka et al., 2005). Interestingly, the TM duplication in TbYme1 has occurred in *T. brucei* only, but not in other kinetoplastids like *Trypanosoma cruzi*, *Leishmania major* or *Bodo saltans*.

In cultured human cells, it was observed that a small subset of mitochondrial proteins display high sensitivity towards heat stress (Wilkening et al., 2018). Among the most aggregation-prone proteins observed were elongation factor Tu (EF-Tu) and a succinate dehydrogenase flavoprotein subunit (SDHA). EF-Tu, in addition to its function in protein translation, possesses chaperone activity and prevents thermal protein aggregation and facilitates protein refolding under stress conditions (Caldas et al., 1998; Rao et al., 2004; Suzuki et al., 2007). In light of the high propensity to form aggregates after heat-stress, the detection of TbSdh5 as interacting proteins of TbYme1 suggests that its role in *T. brucei* may be to clear misfolded mitochondrial protein aggregates, possibly aided by the chaperone function of EF-Tu. This would explain the loss of viability at elevated temperatures in parasites lacking TbYme1. Unlike for the mitochondrial unfolded protein response in other organisms (Shpilka and Haynes, 2018), we did not observe an increase in heat-shock proteins TbHsp60 or TbHsp70 in TbYme1−/− parasites. Whether or not trypanosomes possess canonical mitochondrial quality control systems is not known, however, very recent data indicate the presence of mitochondria-nuclear communication and targeted degradation of mislocalized proteins (Dewar et al., 2021). On the other hand, the turnover of TbPOMP24 was increased in the absence of TbYme1, suggesting a dual role of the TbYme1-TbSlp2 complex in protein stabilization/degradation. TbPOMP24 is a large mitochondrial protein with a C-terminal signal peptide, a single transmembrane domain and a putative protein interaction module used for homo- and hetero-oligomerization, called SAM (sterile alpha motif) domain. The function of TbPOMP24 in *T. brucei* has not been investigated.

In mammalian cells, SLP-2 has been shown to bind to the mitochondrial glycerophospholipid CL, forming CL-enriched membrane domains required for optimal formation and function of protein complexes involved in oxidative phosphorylation (Rivera-Milla et al., 2006; Christie et al., 2011; Mitsopoulos et al., 2015). In contrast, we found that knocking out TbSlp2 in *T. brucei* had no effect on the stability of the respiratory complexes, and on mitochondrial morphology or CL biosynthesis. The human enzymes involved in CL formation assemble into a CL synthesis complex supported by PGS1 oligomers, and the complex interacts with many known CL-binding proteins (Serricchio et al., 2018). In *T. brucei*, TbPgs and TbCls also form a large mitochondrial complex (Serricchio and Bütikofer, 2013). Based on the observation that CL is found in tight association with many mitochondrial protein complexes (Planas-Iglesias et al., 2015) and is rapidly hydrolyzed when floating freely in membranes (Xu et al., 2016), it is tempting to speculate that the CL biosynthesis complex is recruited to sites where CL is needed for efficient incorporation into CL-dependent proteins or protein complexes. In analogy to human cells (Christie et al., 2011; Serricchio et al., 2018), TbSlp2 co-precipitated with both TbPgs and TbPhb1. These interactions suggest that there is a connection between CL biosynthesis and membrane domain formation mediated by multimeric TbPhb and TbSlp2 complexes in *T. brucei*.

## Methods

Unless otherwise stated, all reagents were purchased from Sigma Aldrich (Buchs, Switzerland).

### Trypanosome Cultures

*T. brucei* procyclic form SmOx P9 parasites (Poon et al., 2012) expressing hSpCas9 (Beneke et al., 2017) were cultured at 27°C in SDM79 containing 10% (v/v) heat-inactivated fetal bovine serum, 5 μg/ml blasticidin (InvivoGen), 2 μg/ml puromycin. Knock-out parasites were cultured in presence of additional 15 μg/ml G418 (geneticin; Santa Cruz Biotechnology, Heidelberg, Germany) and 25 μg/ml hygromycin (InvivoGen, Nunningen, Switzerland). TbYme1 addback cultures were selected and cultured in the presence of 150 μg/ml nourseothricin (Jena Bioscience, Jena, Germany).

### Gene Knockout and Tagging With CRISPR/Cas9

Genome editing was achieved following the genome editing toolkit for kinetoplastids (Beneke et al., 2017). Donor resistance cassettes for knock-outs were amplified by PCR from pPOTv6 plasmids (Dean et al., 2015) with hygromycin and geneticin resistance cassettes using primers as described (Beneke et al., 2017). 5 and 3′ short-guide DNAs comprising a T7 promoter sequence, a Cas9 binding site, and a stretch of 20 nucleotide long 5 and 3′ target sequences were PCR-assembled as described (Beneke et al., 2017). Donor cassettes for tagging were amplified from pMOTag vectors (Oberholzer et al., 2006). PCR reactions were performed with Expand High Fidelity PCR System (Roche, Basel, Switzerland) with primers generated by the online tool www.leishgedit.net. PCR reactions were pooled and transfected using the 4D-Nucleofector system (Lonza, Basel, Switzerland). After 4 h recovery, selection antibiotics were added. Control PCRs to verify gene knockout were done with primers TbSlp2_UTR_fwd: GTT​GTT​GGT​GCT​ATT​GTT​GCT​ATG; TbSlp2_UTR_rev: CGCATCCGCCTATGCAA; TbSlp2_ORF_fwd: GTA​GTT​TGG​TGC​ACT​CGT​CTC; TbSlp2_ORF_rev: CCA​ACG​AAG​TGG​CAC​TAA​CC; TbYme1_UTR_fwd: GAG​GGA​TAA​TAC​GAA​GAG​GAG​AAC; TbYme1_UTR_rev: CGT​GTG​CAT​GCT​GCT​TAG; TbYme1_ORF_fwd: CGC​TGT​CCC​ATC​ATA​CCA​G; TbYme1_ORF_rev: CAA​CAA​TAA​CCA​CAG​TCG​GG.

### Preparation of Crude Membrane Fractions

Crude mitochondrial preparations were obtained by digitonin extraction as described (Charriere et al., 2006). Briefly, trypanosomes were washed in TBS (10 mM Tris·HCl pH 7.5, 144 mM NaCl) and suspended in 0.5 ml SoTE (20 mM Tris·HCl, pH 7.5, 0.6 M sorbitol, 0.2 mM EDTA) followed by the addition of 0.5 ml SoTE containing 0.05% (w/v) digitonin. After 5 min on ice, crude membranes were collected by centrifugation (6000 x g, 5 min, 4°C).

### Carbonate Extraction

Crude membrane preparations from 2 × 10^8^ parasites were re-suspended in water containing protease inhibitors (Roche), separated into three individual tubes, and equal volumes of 0.2 M sodium carbonate at pH 10.5, pH 11.5 or pH 12.5 were added. After 1 h of incubation on ice, the membrane fractions were collected by ultra-centrifugation (100’000 x g, 30 min, 4°C), dissolved in 100 µl SDS sample buffer and heated for 5 min at 65°C. Alkaline-solubilized protein fractions were precipitated (Wessel and Flugge, 1984), dissolved in 100 µl SDS sample buffer and heated for 5 min at 65°C.

### Protein immunoprecipitation

Immunoprecipitations were performed as described in (Serricchio et al., 2018) with minor modifications. Log-phase parasites (approx. 10^8^) were collected, washed in TBS and lysed in 900 µl lysis buffer [10 mM Tris-HCl, pH 7.4, 150 mM NaCl, 1 mM EDTA, 1% (v/v) Triton X-100, 0.5% (v/v) NP-40, protease inhibitor cocktail (Roche)]. Alternatively, some experiments were done with lysis buffer containing 2% (w/v) DDM. After clearing the lysate for 10 min at 17′000 x g, an input sample was removed and the lysate distributed into three tubes, to which 1 µg of the following antibodies were added: mouse anti-c-Myc antibody (9E30, Santa Cruz Biotechnology, Heidelberg, Germany), or mouse anti-HA antibody (HA.11, 16B12, Enzo Life Sciences, Lausen, Switzerland). After 2 h rotation at 4°C, Protein G Dynabeads (Invitrogen, Reinach, Switzerland) were added for 16 h. Beads were washed in cold lysis buffer, proteins eluted with SDS sample buffer and heated at 62°C for 10 min.

### Lipid Overlay Assay

To isolate TbSlp2-HA from *T. brucei*, parasites (3.5 × 10^8^) were lysed in 140 µl RIPA buffer [10 mM Tris-HCl, pH 8.0, 140 mM NaCl, 1 mM EDTA, 0.5 mM EGTA, 1% (v/v) Triton X-100, 0.1% (w/v) sodium deoxycholate, 0.1% (w/v) SDS, protease inhibitor cocktail] for 5 min at 65°C, then diluted with 1,260 µl IP buffer [10 mM Tris-HCl, pH 7.4, 150 mM NaCl, 1 mM EDTA, 1% (v/v) Triton X-100, 0.5% (v/v) NP-40, protease inhibitor cocktail (Roche)]. After clearing the lysate at 17’000 x g for 30 min at 4°C, anti-HA agarose (Roche) was added for 16 h at 4°C. After extensive washing with IP buffer, proteins were eluted with 0.1 M glycine (pH 2.5) and dialyzed against PBS (137 mM NaCl, 2.7 mM KCl, 10 mM Na_2_HPO_4_, 1.8 mM KH_2_PO_4_).

To purify GST-TbSlp2, TbSlp2 was cloned into plasmid pGEX-6P-1 and transformed into *Escherichia coli* BL21. Exponentially growing cultures were induced with 0.1 mM IPTG for 3 h at 25°C. Pelleted bacteria were suspended in 40 ml lysis buffer (PBS, pH 7.4, 0.5 mg/ml lysozyme, 1 mM DTT, 2 mM MgCl_2_) and kept on ice for 30 min. After three freeze-thaw cycles, lysates were centrifuged at 12,000 x g for 30 min at 4°C. Glutathione-sepharose 4B (1 ml; GE Healthcare) was added to the cleared lysates and incubated under rotation at 4°C for 60 min. After extensive washing with PBS, beads were equilibrated with equilibration buffer (50 mM Tris-HCl, 2 mM MgCl_2_, pH 8.0). The GST fusion proteins were eluted with freshly prepared equilibration buffer supplemented with 20 mM reduced glutathione. Eluted fractions were analyzed by SDS-PAGE and immunoblotting, pooled and dialyzed against PBS. Lipid overlay experiments were done using Membrane Lipid Strips (Echelon Biosciences, Salt Lake City, United States) according to the manufacturer’s instructions.

### Metabolic Labeling

Parasites were incubated with [^3^H]-glycerol and phospholipids were extracted and analyzed exactly as described before (Serricchio and Bütikofer, 2012).

### Quantification of Reactive Oxygen Species

Parasites were washed in PBS, counted and diluted in PBS to 5 × 10^6^/ml. Aliquots of 100 µl were added per well into 96-well plates. H_2_O_2_ (1 ml) was added to control wells for 5 min, followed by addition of 20 µM dichlorodihydrofluorescein diacetate to all wells. After incubation for 20 min at 27°C, the samples were analyzed using an excitation wavelength of 485 nm and emission wavelength of 530 nm using a Spark Microplate reader (TECAN, Männedorf, Switzerland).

### Generation of TbYme1 Addback Parasites

The TbYme1 ORF was amplified from gDNA using primers SalI_fwd ATA​AGT​CGA​CAT​GCA​CCG​GCG​CTG​TC and Xho_rev AGA​CTC​GAG​CGT​TAT​GGA​AAC​GGG​GCG​TTG (restriction sites underlined), digested and ligated into pGS plasmids (Gottier et al., 2017) containing a C-terminal Myc-tag and a SAT1 resistance cassette under a constitutive procyclin promoter. For mutagenesis, inverse PCR was performed using primers fwd CCA​ATG​CGT​CGA​TTT​GGT​CTA​TAA​AAA​TAA​GCG and rev CGC​TTA​TTT​TTA​TAG​ACC​AAA​TCG​ACG​CAT​TGG.

### Cycloheximide Treatment

Cells were diluted to 0.6 × 10^7^/ml in 5 ml medium and treated with 100 μg/ml cycloheximide. Aliquots (0.9 ml) were removed at each time point, washed in TBS and lysed in 50 µl lysis buffer [10 mM Tris-HCl, pH 7.4, 150 mM NaCl, 1 mM EDTA, 1% (v/v) Triton X-100, 0.5% (v/v) NP-40, protease inhibitor cocktail]. After addition of SDS sample buffer, extracts were heated at 62°C for 5 min.

### Native Polyacrylamide Gel Electrophoresis (Native PAGE) and immunoblotting

Native PAGE was performed with DDM-solubilized crude mitochondrial membrane fractions and separated on 3–12% native PAGE gradient gels (Invitrogen Reinach, Switzerland) at 4°C (Wittig et al., 2006). Proteins were transferred onto nitrocellulose membranes (Thermo Scientific) using a semi-dry protein blotting system (BioRad, Cressier, Switzerland). After blocking in TBS containing 5% (w/v) milk powder, membranes were exposed to primary antibodies rabbit anti-Cox4, rabbit anti-Cyt c1, rabbit anti-ATP synthase subunit β, or mouse anti-Hsp60 antibody (kindly provided by André Schneider, University of Bern, Bern, Switzerland), diluted 1:1,000 in TBS containing 5% (w/v) milk powder. Horseradish peroxidase-conjugated (HRP) anti-mouse, anti-rabbit (Dako, Glostrup, Denmark), anti-HA (HA.11, 16B12, Enzo Life Sciences, Lausen, Switzerland) or anti-cMyc (Santa Cruz Biotechnology) were used at dilutions of 1:5,000, and detected using an enhanced chemiluminescence detection kit (Thermo Scientific). Protein sizes were determined using NativeMark™ Unstained Protein Standard (Invitrogen, Reinach, Switzerland). Bands on blots were quantified using the gel analyzer function of Fiji (Schindelin et al., 2012). Signal intensities of multiple blots and different exposure times were analyzed with the multiple *t*-test function in GraphPad Prism software (Version 6.0 g).

### Immunofluorescence Microscopy

Parasites at a cell density of 10^6^ in mid-log growth phase were washed and suspended in PBS, allowed to adhere on a microscope slide (Thermo Scientific) for 15 min and fixed in PBS containing 4% (w/v) paraformaldehyde for 10 min. After washing and permeabilization for 5 min with PBS containing 0.1% (w/v) Triton X-100, cells were blocked for 30 min with 2% (w/v) bovine serum albumin in PBS. The following antibodies were diluted 1:250 in blocking solution and added to cells for 30 min: mouse anti-c-Myc IgG (c-Myc 9E30; Santa Cruz Biotechnology, Heidelberg Germany), mouse anti-HA (HA.11, 16B12; Enzo Life Sciences, Lausen, Switzerland), rabbit anti-TbACC (provided by Alena Ziková, Biology Centre of the Czech Academy of Sciences) (Pena-Diaz et al., 2012), or mouse anti-Hsp70 (provided by André Schneider, University of Bern, Bern, Switzerland). After washing, cells were incubated for 30 min with goat anti-mouse Alexa Fluor 488 or goat anti-rabbit Alexa Fluor 594 (1:500 in blocking solution), washed again and air-dried before mounting with Vectashield (Vector Laboratories, Burlingame, CA) containing 1.5 μg/ml 4’,6-diamidino-2-phenylindole (DAPI). The images were acquired using a Leica DMI6000 B microscope with ×60 oil objective.

### Seahorse Flux Analysis

Parasites were counted, washed twice in fetal bovine serum-free SDM79 and diluted to a density of 5.5 × 10^6^/ml. Aliquots (180 μl; 10^6^ cells) were added to the wells of an 8-well microplate pre-treated with Cell-Tak (Corning), as described elsewhere (Shah-Simpson et al., 2016). The plate was centrifuged for 2 min at 300 x g and immediately analyzed using a Seahorse XFp Flux analyzer (Agilent Technologies). Parasites were still adhered to the bottom and motile after the experiments. Data were analyzed using Wave software (version 2.6.1, Agilent Technologies).

## Data Availability

The original contributions presented in the study are included in the article/Supplementary Material, further inquiries can be directed to the corresponding author.
