# Supplementary material for: A Conserved Mitochondrial Chaperone-Protease Complex Involved in Protein Homeostasis
Source: Front Mol Biosci. 2021 Nov 9;8:767088. doi: 10.3389/fmolb.2021.767088 (PMC8630662; doi:10.3389/fmolb.2021.767088)
Supplement: Supplementary file 1 [file DataSheet1.PDF]

Supplementary Material

1.1 Supplementary Figures

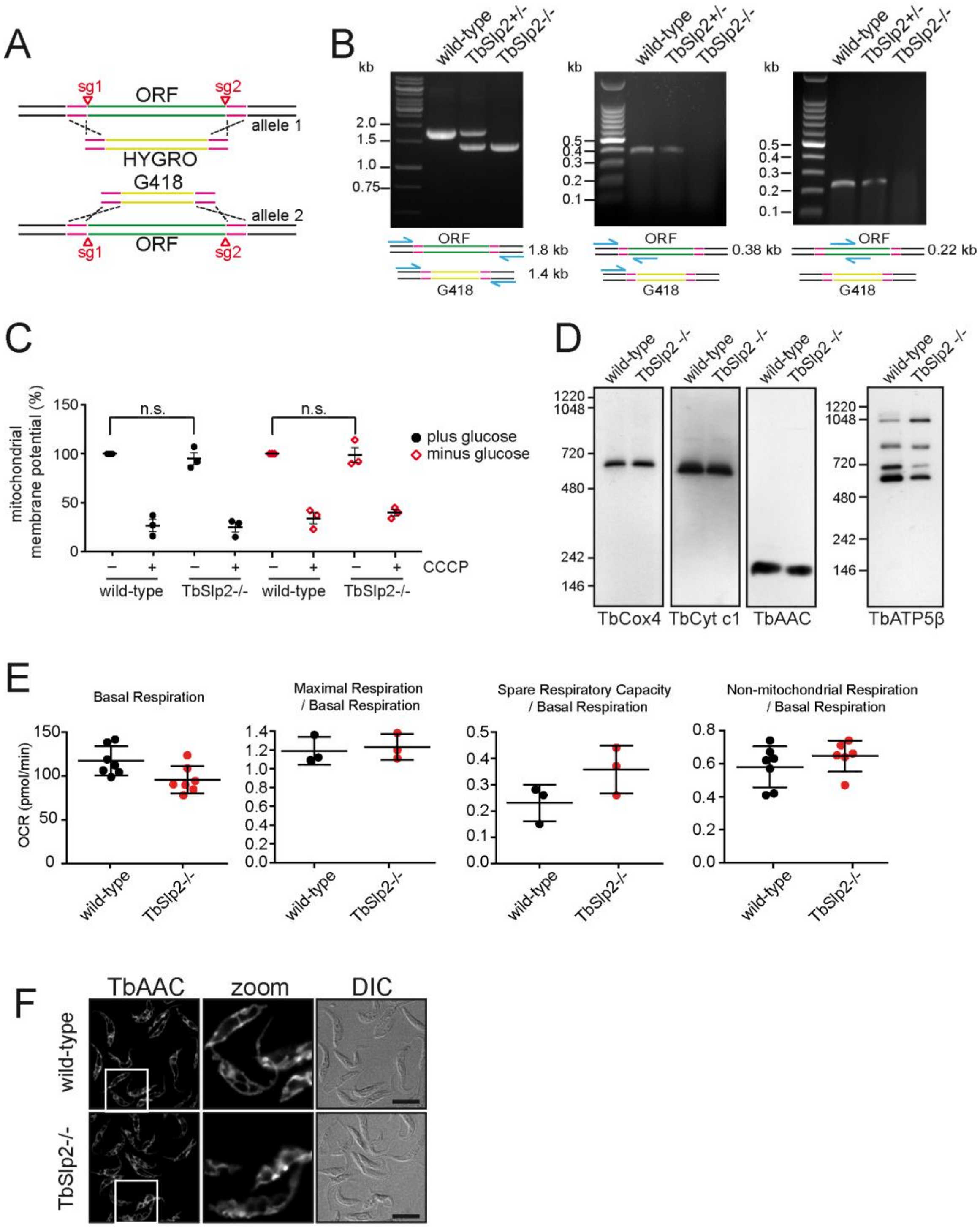

Supplementary Figure 1. Characterization of TbSlp2-/- parasites. A) Strategy to generate TbSlp2 knock-out (TbSlp2-/-) parasites using Crispr/Cas9. sgDNA-targeted double-strand breaks flanking the open-reading frame (ORF) was followed by template-driven integration of G418 or hygromycin resistance genes, respectively. B) Antibiotic-resistant clones were collected and tested by PCR. The parental cells and two clones were tested with primers up- and downstream of the ORF (left panel), with primers binding in the 5`-untranslated region and within the ORF (right panel), and with a primer pair binding within the ORF (right panel). A TbSlp2-/- clone was generated using a single resistance cassette, which was integrated into both ORFs simultaneously. C) The mitochondrial membrane potential was quantified using tetramethylrhodamine ethyl ester (TMRE) staining and measurement of fluorescence intensity using a 96-well plate reader. Parasites were cultured in regular SDM79 containing glucose or adapted to glucose-free medium. Carbonyl cyanide m-chlorophenylhydrazone (CCCP) was added to disrupt the mitochondrial membrane potential. n.s.: not significant. D) Native-PAGE and immunoblot analysis of complex IV (stained with TbCox4), complex III (TbCyt c1), complex V (ATP synthase subunit β or TbAAC in wild-type and TbSlp2-/- parasites. E) Oxygen consumption measured with a Seahorse XFp Analyzer. Wild-type and TbSlp2-/- cells were adhered to the bottom of the wells using Cell-Tak and oxygen consumption rate (OCR) was measured after addition of oligomycin A, CCCP and rotenone/antimycin. Obtained values are given relative to basal respiration to reduce dependence from absolute cell numbers. F) Immunofluorescence microscopy of wild-type and TbSlp2-/- parasites stained with an antibody against TbAAC. Scale bar: 10 µm.

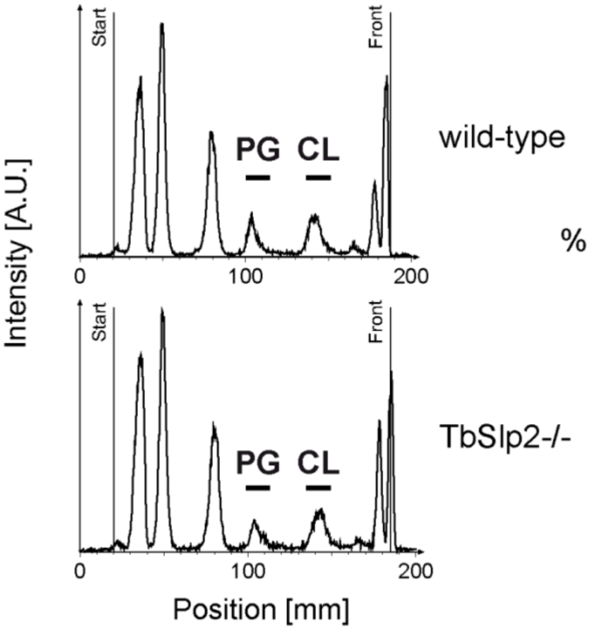

% label	wild-type	TbSlp2-/-
PG	7.8 ± 0.3	7.0 ± 0.2
CL	11.5 ± 0.7	11.1 ± 0.2

Supplementary Figure 2. Depletion of TbSlp2 does not affect *de novo* cardiolipin biosynthesis. Wild-type and TbSlp2-/- parasites were cultured in presence of 10 µCi [^{3}H]-glycerol for 4 h. Cells were washed, lipids were extracted, separated by thin-layer chromatography and analyzed by radioisotope scanning. Peak of peaks representing PG and CL were quantified (n=3).

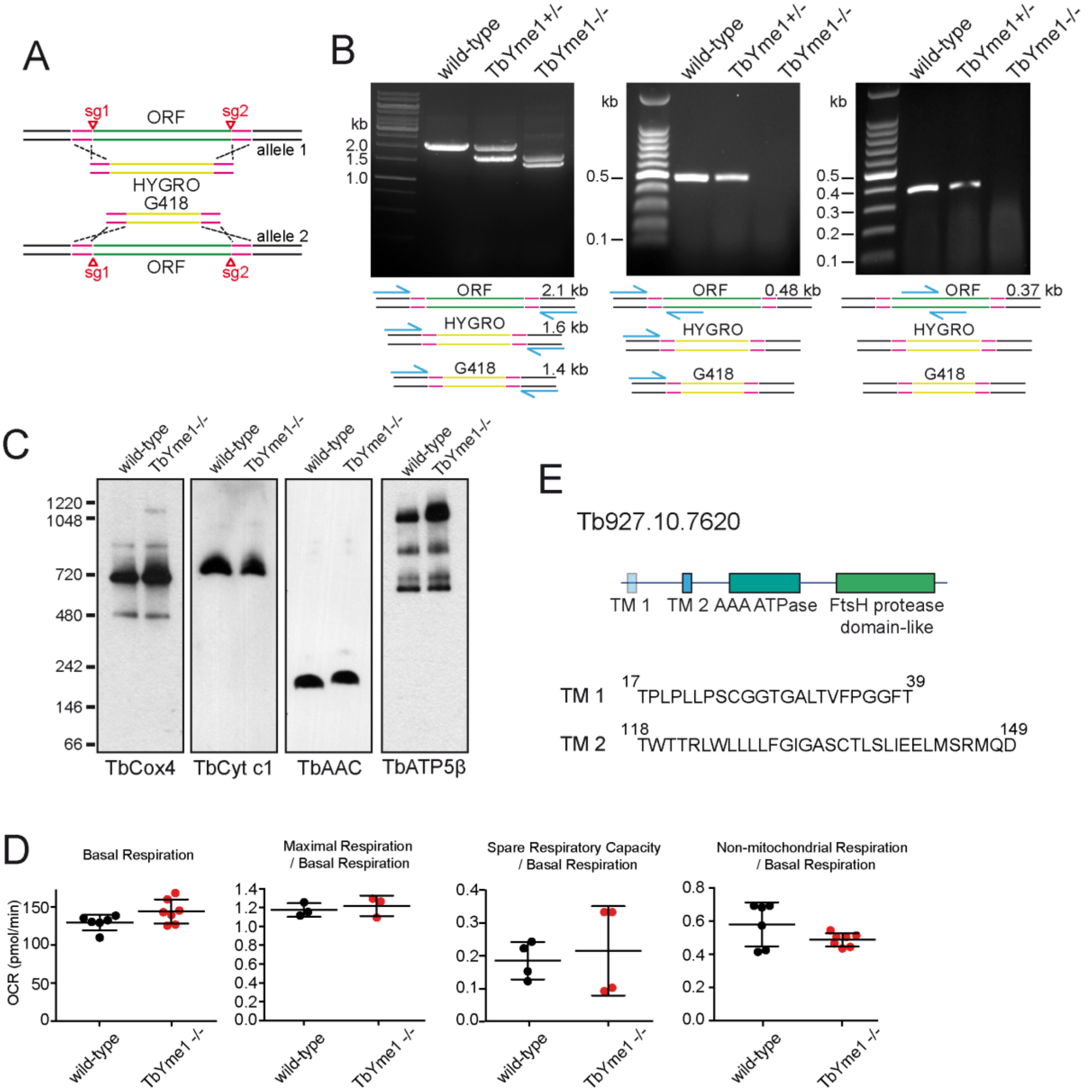

Supplementary Figure 3. Characterization of TbYme1-/- clones. A) Strategy to generate gene knock-out using Crispr/Cas9. sgDNA-targeted double-strand breaks flanking the open-reading frame (ORF) followed by template-driven integration of G418 or hygromycin resistance genes, respectively. B) After transfection, antibiotic-resistant clones were collected and tested by PCR. The parental culture and two clones were tested with primers up- and downstream of the ORF (left panel). Moreover, parental cells and the two clones were tested with primers binding upstream and within the ORF (right panel). In addition, the absence of the ORF was verified with a third primer pair binding within the ORF (right panel). C) Native-PAGE and immunoblot analysis of complex IV (stained with TbCox4), complex III (TbCyt c1), TbAAC or complex V (ATP synthase subunit β) in wild-type and TbYme1-/- parasites. D) Oxygen consumption measured with a Seahorse XFp analyzer. Wild-type and TbYme1-/- cells were adhered to the bottom of the wells using Cell-Tak and oxygen consumption was measured after addition of oligomycin A, CCCP and rotenone/antimycin. Obtained values are given relative to basal respiration to reduce dependence from absolute cell numbers. E) Amino acid sequences of the two predicted transmembrane domains of TbYme1.

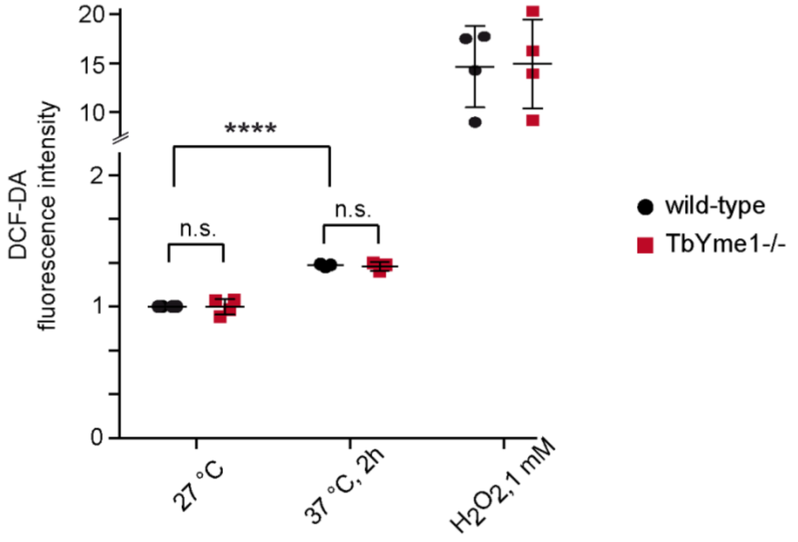

Supplementary Figure 4. Quantification of ROS levels. Reactive oxygen species (hydroxyl, peroxyl and other reactive oxygen species) were quantified using dichlorodihydrofluorescein diacetate (DCFH-DA). Parasites cultured at 27 °C or after a 2 h heat-pulse at 37 °C were quantified. Addition of 1 mM H2O2 was used as a positive control. n.s.: not significant. ****: $p < 0.0001$

1.2 Supplementary Tables

Name	Protein_ID	unique peptides	sequence coverage [%]	Mol. weight [kDa]	fold change [log2]	adjusted p-value
TbSlp2	Tb927.5.520	30	58.8	56	11.2	8.5E-44
TbPOMP24 *	Tb927.3.3130	18	20.6	178	7.8	2.5E-05
TbYme1	Tb927.10.7620	64	71.4	71	7.6	1.7E-22
TbMSP-B, putative	Tb927.8.1630	18	56.3	60	7.5	9.7E-22
hypothetical protein, conserved	Tb927.3.4480	5	57.4	17	6.2	8.0E-16
hypothetical protein, conserved	Tb927.10.3290	6	42.9	31	6.2	1.5E-15
hypothetical protein, conserved	Tb927.9.5000	10	48.5	31	5.8	6.2E-14
zinc finger protein family member, putative	Tb927.7.2670	6	20.0	50	5.4	1.3E-12
prohibitin 1	Tb927.8.4810	5	22.4	31	5.0	8.7E-11
prohibitin 2	Tb927.10.4310	7	34.9	32	4.6	1.6E-09
succinate dehydrogenase flavoprotein subunit, putative (TbSdh5) *	Tb927.3.3460	3	11.4	53	4.5	2.8E-04
hypothetical protein, conserved	Tb927.10.7870	4	29.8	23	4.4	1.1E-08
Elongation factor Tu, mitochondrial	Tb927.10.13360	6	16.2	52	4.2	3.3E-08
hypothetical protein, conserved	Tb927.6.4240	5	52.9	21	4.1	5.7E-08
mitochondrial processing peptidase, beta subunit, putative	Tb927.5.1060	4	18.0	54	3.9	3.7E-07
TbMCP11	Tb927.9.10310	4	21.8	34	3.8	5.6E-07
metallo-peptidase, Clan ME, Family M16	Tb927.11.3980	6	21.0	57	3.6	1.9E-06
succinyl-CoA ligase beta-chain, putative	Tb927.10.7410	4	16.0	45	3.0	6.2E-05
hypothetical protein, conserved	Tb927.10.12930	2	15.0	24	3.0	7.7E-05
cytochrome oxidase subunit IV (TbCox4)	Tb927.1.4100	2	9.1	41	2.8	2.4E-04
chaperone protein DnaJ, putative	Tb927.7.990	3	5.7	87	2.3	2.8E-03
chaperone protein DnaJ, putative	Tb927.9.12730	2	5.8	49	1.7	2.4E-02
TbMCP13	Tb927.2.2970	2	10.3	34	1.7	2.8E-02
cytochrome oxidase subunit X (TbCox10)	Tb927.11.13140	2	23.3	14	1.3	9.9E-02
hypothetical protein, conserved	Tb927.2.5860	2	8.7	134	1.0	2.1E-01

Supplementary Table 1. Putative mitochondrial TbYme1 substrates. Mitochondrial proteins that interact with TbYme1-E286Q. The list is sorted according to [log2] fold change. * Those proteins were identified only and exclusively after a heat pulse of 37 °C for 2 hours. N = 3; for the heat-pulse experiments N = 2.
